# Supplementary material for: Drug repurposing for aging research using model organisms
Source: Aging Cell. 2017 Jun 16;16(5):1006–15. doi: 10.1111/acel.12626 (PMC5595691; doi:10.1111/acel.12626)
Supplement: Supplementary file 7 — Data S1 Zip‐Archive of all report cards. [file ACEL-16-1006-s007.zip › RC_GLU.pdf]

GLU

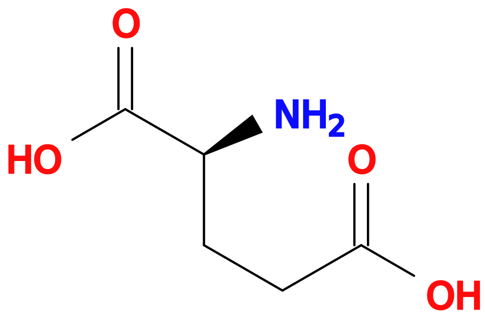

Database identifiers

ChEMBLCompound	CHEMBL575060
DrugBank	DB00142
DrugBank	DB02517
CHEBI	16015
eMolecules	477277

Ranking

	Rank	Score
Drosophila	512/697	0.258
C. elegans	288/591	0.199

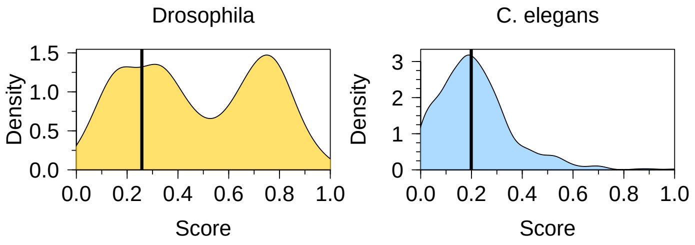

	Ageing implication	Domain conservation	Binding site conservation	Binding affinity	Bioavailability	Lipinski	Promiscuity	Purchasability	Drug approval	**Total**
Drosophila	0.36	0.582	0.896	0.494	(0.9)	0.0	-0.0	0.1	0.075	0.258
C. elegans	0.36	0.727	0.896	0.494	0.212	0.0	-0.0	0.1	0.075	0.199

Names

- **L-glutamic acid**
- (S)-2-aminopentanedioic acid
- D-Glutamic Acid
- E
- Glu
- Glutamate
- L-Glutaminsaeure

Roles

nutraceutical, micronutrient, neurotransmitter

Status

Approved drug (according to ChEMBL)	No
Classification (according to DrugBank)	experimental
Number of Rule of 5 violations	0
Binding affinity to original target in log units (RF-Score prediction)	4.98
Burns *C. elegans* bioavailability prediction	-5.35

Compound Target Characteristics

Glutamate–cysteine ligase

Best gene implication in ageing for this target family came from gene A4V448 via mapping the annotation from Ensembl FBgn0040319 via mapping the annotation from EntrezGene 53581 via mapping the annotation from GenAgeModels 0293 annotated in GenAge release 17.

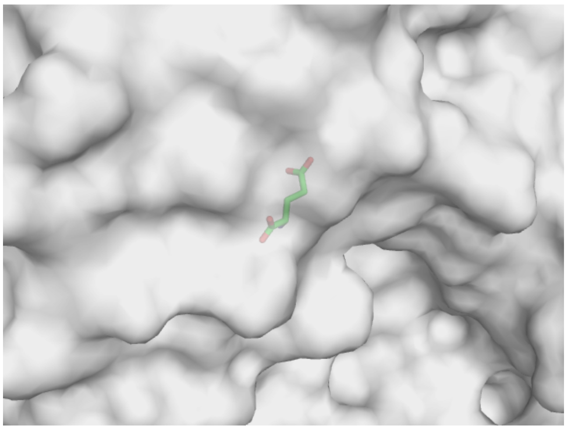

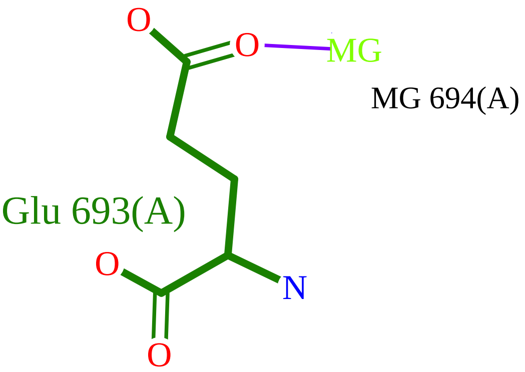

protein	amino acids contacts (binding site)										
PDB:3ig5:chainA:P32477	E	E	C	S	C	Q	R	I	Y	W	R
PDB:3ig8:chainA:P32477	E	E	C	S	C	Q	R	I	Y	W	R
sp:P48506:GSH1_HUMAN	E	E	N	C	C	Q	R	I	Y	W	R
tr:Q14TF0:Q14TF0_HUMAN	E	E	N	C	C	Q	R	I	Y	W	R
sp:P19468:GSH1_RAT	E	E	N	C	C	Q	R	I	Y	W	R
tr:Q3TEF1:Q3TEF1_MOUSE	E	E	N	C	C	Q	R	I	Y	W	R
sp:P97494:GSH1_MOUSE	E	E	N	C	C	Q	R	I	Y	W	R
tr:A4V448:A4V448_DROME	E	E	C	C	C	Q	R	I	Y	W	R
sp:Q9W3K5:GSH1_DROME	E	E	C	C	C	Q	R	I	Y	W	R
sp:Q20117:GSH1_CAEEL	E	E	C	C	C	Q	R	I	Y	W	R
tr:E9P9G0:E9P9G0_YEASX	E	E	C	S	C	Q	R	I	Y	W	R
tr:E9P9M0:E9P9M0_YEASX	E	E	C	S	C	Q	R	I	Y	W	R
tr:F5CIK1:F5CIK1_YEASX	E	E	C	S	C	Q	R	I	Y	W	R
sp:P32477:GSH1_YEAST	E	E	C	S	C	Q	R	I	Y	W	R

protein	whole protein ident	simil	domain-based ident	simil	contact-based ident	simil
PDB:3ig5:chainA:P32477	1.0	1.0	1.0	1.0	1.0	1.0
PDB:3ig8:chainA:P32477	1.0	1.0	1.0	1.0	1.0	1.0
sp:P48506:GSH1_HUMAN	0.4	0.72	0.41	0.72	0.82	0.77
tr:Q14TF0:Q14TF0_HUMAN	0.4	0.72	0.41	0.72	0.82	0.77
sp:P19468:GSH1_RAT	0.41	0.72	0.41	0.72	0.82	0.77
tr:Q3TEF1:Q3TEF1_MOUSE	0.41	0.72	0.41	0.72	0.82	0.77
sp:P97494:GSH1_MOUSE	0.41	0.72	0.41	0.72	0.82	0.77
tr:A4V448:A4V448_DROME	0.33	0.62	0.36	0.63	0.91	0.9
sp:Q9W3K5:GSH1_DROME	0.33	0.62	0.36	0.63	0.91	0.9
sp:Q20117:GSH1_CAEEL	0.34	0.68	0.37	0.7	0.91	0.9
tr:E9P9G0:E9P9G0_YEASX	1.0	1.0	1.0	1.0	1.0	1.0
tr:E9P9M0:E9P9M0_YEASX	1.0	1.0	1.0	1.0	1.0	1.0
tr:F5CIK1:F5CIK1_YEASX	1.0	1.0	0.99	1.0	1.0	1.0
sp:P32477:GSH1_YEAST	1.0	1.0	1.0	1.0	1.0	1.0

Gclc (FBgn0040319) associated phenotypes

chemical resistant, long lived

(Information from FlyBase)

Gclc (UniProt:Q9W3K5) annotation

Pathway: Sulfur metabolism; glutathione biosynthesis; glutathione from L-cysteine and L-glutamate: step 1/2.

(Information from UniProt)

gcs-1 (UniProt:Q20117) annotation

Pathway: Sulfur metabolism; glutathione biosynthesis; glutathione from L-cysteine and L-glutamate: step 1/2.

(Information from UniProt)
